# Monitoring health and reproductive status of olms (*Proteus anguinus*) by ultrasound

**DOI:** 10.1371/journal.pone.0182209

**Published:** 2017-08-15

**Authors:** Susanne Holtze, Maja Lukač, Ivan Cizelj, Frank Mutschmann, Claudia Anita Szentiks, Dušan Jelić, Robert Hermes, Frank Göritz, Stanton Braude, Thomas Bernd Hildebrandt

**Affiliations:** 1 Department of Reproduction Management, Leibniz Institute for Zoo and Wildlife Research, Berlin, Germany; 2 Department of Poultry Diseases with Clinic, Faculty of Veterinary Medicine, University of Zagreb, Zagreb, Croatia; 3 Zagreb Zoo, Zagreb, Croatia; 4 Exomed – Institut für veterinärmedizinische Betreuung niederer Wirbeltiere und Exoten GbR, Berlin, Germany; 5 Department of Wildlife Diseases, Leibniz Institute for Zoo and Wildlife Research, Berlin, Germany; 6 Croatian Institute for Biodiversity, Zagreb, Croatia; 7 Department of Biology, Washington University in St. Louis, St. Louis, Missouri, United States of America; University of Missouri Columbia, UNITED STATES

## Abstract

The olm (*Proteus anguinus*) is a troglomorphic, neotenous amphibian with extraordinary life expectancy and unique adaptations that deserve further investigation. A low reproductive rate and habitat decline render it threatened by extinction. Establishing captive populations for maintenance and artificial breeding may one day become crucial to the species. Longitudinal, *in-vivo* assessment of inner organs is invaluable to our understanding of reproductive physiology, health, and behavior. Using ultrasound, we measured heart rate and assessed health and reproductive status of 13 captive olms at Zagreb Zoo. Heart rate averaged 42.9 ± 4.6 bpm (32–55 bpm), as determined via pulsed-wave Doppler at 4–12 MHz. By using frequencies of up to 70 MHz (ultrasound biomicroscopy), inner organs were visualized in detail. Assessment of the gastrointestinal tract provided insights into feeding status and digestive processes. Several subclinical pathologies were detected, including biliary sludge, subcutaneous edema, ascites, and skin lesions. Detection of skin lesions by ultrasound was more sensitive than visual adspection. Olms with ultrasonographically detected skin lesions tested positive for *Saprolegnia* and were treated. Three of the four affected individuals survived and subsequently tested negative for *Saprolegnia*. Sex was reliably determined; only one individual proved male. The reason for this extreme female-biased sex-ratio remains unknown. However, as most of the individuals were flushed from the caves by strong currents in spring, the sample may not be representative of natural populations. In female olms, different stages of ovarian follicular development were observed with diameters ranging between 0.1 and 1.1 mm. Results were confirmed by comparing ultrasound, necropsy, and histological findings of one dead specimen. In summary, ultrasound proved a valuable tool to support conservation and captive breeding programs by allowing non-invasive assessment of physiological parameters, clinical condition, and reproductive status in olms.

## Introduction

**Fig 1:**
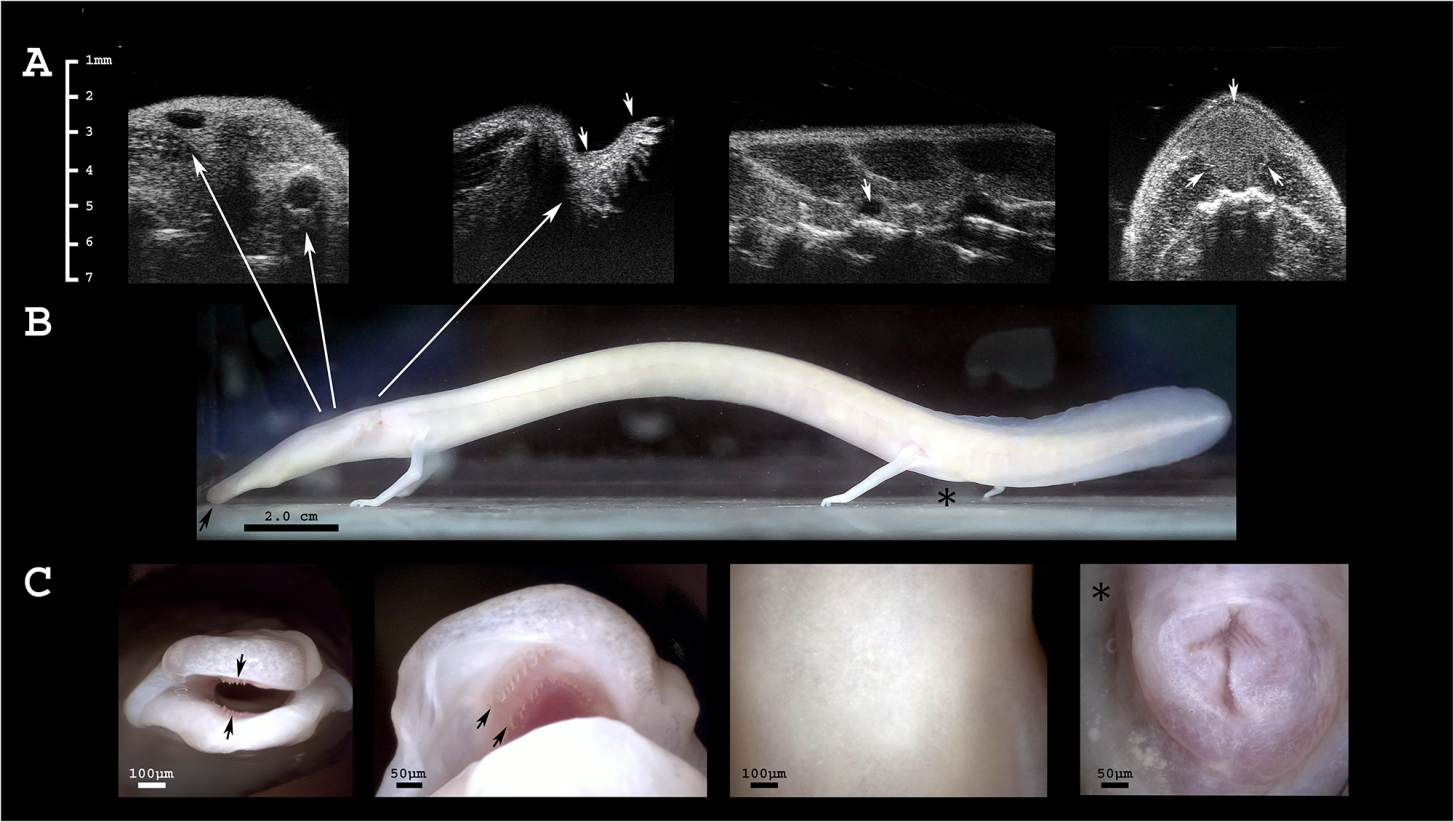
The olm’s gross anatomical characteristics. **A** Ultrasonographic images of the head. From left to right: (i) diagonal section including both, eye and ear of the dead olm (assessment in live specimens is difficult); the ellipsoid shaped eyes are located laterally on the head ca. 0.4 mm beneath the skin surface and appear anechogenic; the lens is visible as a central hyperechogenic line. The ears are composed of the inner ear and a straight canal that distally slightly widens terminating directly beneath the skin surface as circular ring. (ii) Gills in transverse section, arrows indicate their proximal and distal ends; (iii) sagittal section of the body; anechogenic, approximately spherical lymph heart located dorsally of the vertebral column; and (iv) dorsal fat depots in transverse section, indicated by arrows. **B** Photograph of an adult olm in lateral view; head left, tail on the right; the snout is indicated by a black arrow; approximate positions of the ear, eye and gills are indicated by arrows; the position of the cloaca is denoted by a black asterisk. **C** Digital microscope images, from left to right: (i) frontal view of the snout, black arrows indicate the most distal row of teeth in the upper and lower jaw; (ii) black arrows indicate two premaxillary and vomerine teeth, respectively, visible in the upper jaw; (iii) dorsal skin; (iv) cloaca of a female olm; proximal part at the upper side of the image, distal one lower.

Currently, amphibians are the vertebrate class with the largest proportion of species threatened with extinction [1]. Among these is the olm (*Proteus anguinus*), the only representative of the *Proteus* genus and the only purely cave-dwelling vertebrate in Europe. Olms are slender amphibians (Fig 1) with elongated head, rounded snout, and thin extremities. Adapted to a life in complete darkness [2], this species displays various troglomorphic characteristics. The poorly developed eyes are buried beneath the skin, yet mediate responses to light [3–5]. The naturally translucent skin darkens when exposed to light [6]. The species is neotenic, having retained three pairs of external gills and an entirely aquatic life-style; attempts to artificially induce metamorphosis have failed [7].

Olms are endemic to fresh water biotopes of the Dinaric karst, spanning from Northern Italy and Southern Slovenia to South-Western Croatia, Bosnia, and Herzegovina [8]. A critical dependence on clean water makes them susceptible to human impact. Spreading of urban areas, landfills on cave entrances, and water pollution [9,10] have caused them to be classified as vulnerable by the International Union for Conservation of Nature (IUCN) [1]. Although several laws have been implemented to protect this species, populations across the distribution range continue to decline [11].

With an average lifespan of 68.5 years and a predicted maximum lifespan of more than 100 years [12], olms are one of the longest-lived amphibian species. This is unexpected, given its small body size. Despite an extended reproductive period of over 30 years [13], overall reproductive rate is very low. Breeding individuals, eggs, and larvae are rarely found in captivity and, so far, have never been observed in the wild. The most detailed description of reproductive biology is given by Voituron et al. (2010) of an olm population in a semi-natural cave habitat in the French Pyrenees [12]. They found olms to reach sexual maturity very late, on average at an age of 15.6 ± 3.1 years. Olms lay eggs at remarkably long intervals averaging 12.5 ± 0.01 years. Reproductive success is further reduced by relatively small clutch size (35.2 ± 1.8 eggs) and a low hatching rate (0.4 ± 0.01). Males and females of this typically oviparous species [14,15] are monomorphic; sex determination by external traits is not feasible [16].

As with many amphibian species, captive assurance populations and conservation breeding may become necessary to save the species in the future. Assurance populations allow us to manage a species in terms of genetic diversity, to maintain and preserve healthy populations, and to reintroduce offspring back into the wild [17]. Ex situ management has been established for approximately 36 urodele (salamander and newt) species [18]. Captive breeding and release programs for endangered urodeles include *Ambystoma mexicanum*, *Ranodon sibiricus*, *Echinotriton chinhaiensis*, *Calotriton amoldi*, and *Euproctus platycephalus* [18]. The use of ultrasound in amphibians has proven useful for sex determination (e.g. [19]), an essential prerequisite for successful captive management and breeding. For salamanders, this has been limited to a few large species such as the Chinese giant salamander (*Andrias davidianus*) [20] and the hellbender (*Cryptobranchus alleganiensis*) [21]. Assessing sex with ultrasound can be challenging in individuals whose gonads are inactive (e.g. in juveniles or out of breeding season), as well as in small species to which the technique is rarely applied [22]. Few reports exist on the use of ultrasound as a medical diagnostic tool in amphibians [23–25], and none regarding the assessment of heart rate.

Probably owing to their inaccessible habitat, many aspects of olm biology are poorly understood. A deeper knowledge of physiology, health, and reproductive biology will be of great importance for future protection and conservation of the species. We used ultrasonography in olms to non-invasively monitor heart rate, health, reproductive status and to determine sex by imaging the gonads. This work demonstrates the value of ultrasonography as practical tool for basic research, as well as for clinical and captive management application.

## Material and methods

### Animals

We examined all 13 olms kept at the Zoological Garden of Zagreb in 2013. Five of these individuals were captured from natural cave habitat, while eight were rescued after being flushed out of caves by strong currents during the alpine snow-melt in spring. Brief ultrasound examinations (≤ 15 min each) were performed in both summer and autumn of 2013. All of the specimens were of unknown age and sex and were derived from five different locations across Croatia (Table 1).

**Table 1:** Location, number, and origin of captive *Proteus anguinus* in this study.

Location		Number of animals	Origin
Vedrine	Sinj	6	Flushed from caves
Istria	Fontana	2	Flushed from caves
	Picinova cave	2	Captured from natural habitat
Gorski kotar	Rupečica	1	Captured from natural habitat
	Markarova cave	2	Captured from natural habitat

Olms were housed individually in 60 l aquaria under conditions similar to their natural habitat. Air and water temperatures ranged between 8–12°C, and 9–12°C, respectively. Except during feeding, cleaning, and regular health monitoring, animals were kept in complete darkness. Every aquarium contained an air stone (Tetra AS 40 and APS 300, Melle, Germany). Two transparent hiding places enabled visual monitoring of food consumption and health. Water conductivity in the original cave habitat measured 540 μS/cm (Boeco CT-470, Hamburg, Germany). To reach comparable values, tap water was allowed to rest for two weeks. After it was aerated and cooled, it replaced 20% of the water in each tank, every two weeks. Animals were fed at two-week intervals with *Oligochaeta spp*., *Tubifex spp*., *Chironomus spp*., and *Gammarus spp*.

In aquatic animals, the surrounding water serves as a sound propagating medium between ultrasound probe and the object of interest. By performing the examination without direct skin contact, there was no need for sedation or physical restraint. Consequently, handling and stress were minimized. Short manipulation was necessary for transfer to a small weighing basin, placed on an electronic precision scale (Kern KB 240-3N, Kern & Sohn GmbH, Bailingen, Germany). The animal was then transferred to another basin containing an examination chamber (40 x 2 x 2 cm) made of a material with special acoustic properties (Sonokit Proxon, Mediason GmbH, Bad Camberg, Germany) to reduce reflection artifacts. Both, weighing and examination basins were pre-filled with water from the aquarium of the respective animal and were thoroughly disinfected between successive examinations. Animals were returned to their aquaria immediately after examination.

This research study and captive propagation were approved by the Ministry of Environment and Nature Protection of Croatia (UP/I-612-07/15-48/119, 517-07-1-1-1-15-04; UP/I-612-07/11-33/0075, 532-08-01-01-01/1-11-02, respectively). This study was also approved by the *Internal Committee for Ethics and Animal Welfare of the IZW* (approval no. 2013-01-03).

### Basic physiology, health assessment, sex determination, and gonadal development

To assess heart rate, all 13 animals were examined using ultrasonography at frequencies of 4–12 MHz (Voluson I, GE healthcare, Vienna, Austria). One to six pulsed-wave Doppler measurements were obtained per individual; repeated measurements were averaged. Examinations were performed in summer of 2013 and took approximately 10–15 min per animal. During these examinations, heart and gall bladder size were also measured.

Shortly before the second ultrasound examination was performed in autumn, two olms had died of fungal infection. Therefore, we were able to assess health, sex, and gonadal status, only for the remaining eleven live olms. We also conducted ultrasonography on one of the deceased individuals that had been stored at -20°C. For examination of detailed tissue properties and characterization of organ structures of such small organisms, we employed ultrasound biomicroscopy at frequencies of 30–70 MHz (Vevo 2100 and transducer MS700, Visualsonics, Toronto, Canada). This allows for a spatial resolution of up to 30 μm. The full ultrasound examination of the large internal organs (heart, liver, gall bladder, aorta, gastrointestinal tract, lung, gill size, kidney, skin, dorsal muscles, and gonads) took approximately 10–15 min. In addition, we were able to examine eyes, ears, brain, dorsal lymph hearts, and bladder in several individuals, but frequent movements prevented the consistent localization of such small, or ultrasonographically poorly contrasting, organs. Thyroid glands, spleen, fat bodies, and pancreas were not reliably detected, and are not included with our results.

### Necropsy and histology

Reference data for ultrasound examinations in olms do not exist. Consequently, we performed ultrasound, necropsy, and histology of the large inner organs (heart, liver, gastrointestinal tract, lung, kidney, skin, dorsal muscles, and gonads) of one deceased female to verify our sonographic findings. Organ appearance was documented using a digital photo-microscope (Keyence VHX-1000, Osaka, Japan); organ size was measured *post-hoc* on scaled digital images to confirm the accuracy of ultrasound measurements. Also, scaled photographs of the large organs were taken. Organ samples collected during necropsy were fixed in 4% neutral buffered formalin and embedded in paraffin wax for histology. Testes of two male olms, obtained from the German Herrmann cave and preserved in formalin, were included for histological examination. Sections (5 μm thickness) were stained with haematoxylin and eosin (HE). Moreover, recuts of skin and liver were stained with Grocott Methenamine Silver (GMS), Rhodamine, extended Ziehl-Neelsen stain, and Prussian Blue, respectively. Histological images were recorded via a microscope (Zeiss Axioplan, Jena, Germany) with a digital camera (Olympus UC30, Tokyo, Japan).

### Statistics

Results are presented as mean ± standard deviation (SD). Statistical testing of correlation and two-sided Wilcoxon signed-rank tests for paired samples were performed using R-3.2.2 (http://www.r-project.org). Significance threshold of p < 0.05 was used.

## Results

All subjects were of unknown age, possessed fully developed limbs and had body masses of 14.2 ± 5.2 g (range 7.2–24.2 g). Body mass correlated closely with ultrasonographically measured length of vertebrae, diameter of the dorsal aorta, kidney, skin, and dorsal muscles, as well as heart width (r^2^ ≥ 0.74, p ≤ 0.01).

### Heart rate

**Fig 2:**
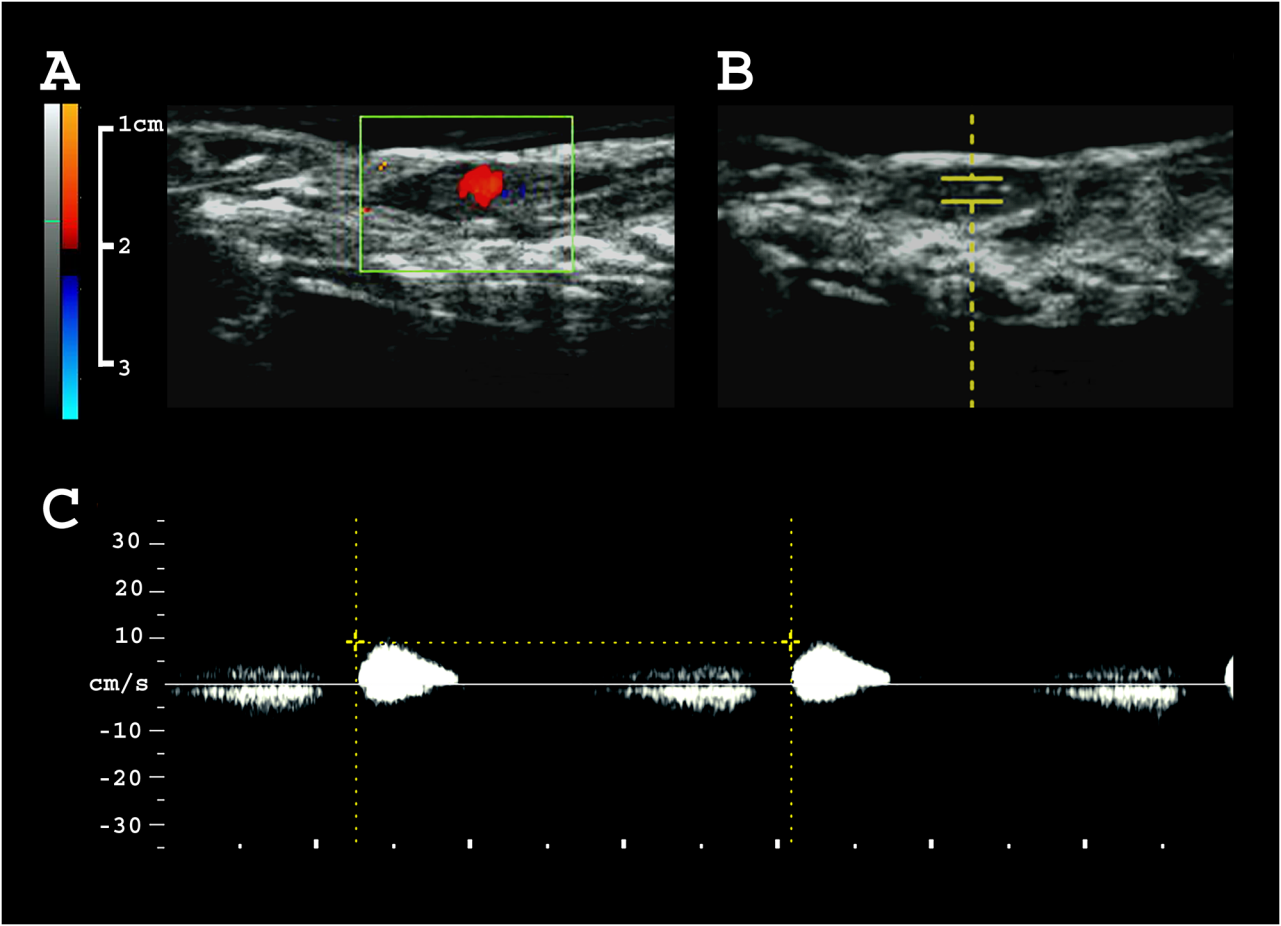
Measuring heart rate by ultrasonography. **A** 2D color Doppler image of the beating heart. **B** B-mode image indicating the localization of pulsed wave Doppler acquisition. **C** Pulsed wave Doppler of the beating heart.

Heart rate of 13 olms, assessed via pulsed-wave Doppler (Fig 2), measured 42.9 ± 4.6 bpm (range 32–55 bpm). Successive measurements, (3.1 ± 1.6 per individual) were reproducible with an intra-individual variation of 1.8 ± 2.1 bpm (maximum deviation = 6 bpm). We found no tendency towards an increase or decrease in heart rate over the course of the examination period. Also, there was no clear correlation between heart rate and body mass (r^2^ = 0.06, p = 0.85) or heart size (r^2^ = -0.36, p = 0.23 for length, and r^2^ = -0.08, p = 0.78 for diameter), respectively. In color Doppler mode, blood flow appears orderly without major turbulences during each heart beat (S1 Video). The dorsal aorta presented slightly hyperechogenic compared to the adjacent liver. Blood flow velocity was not measured, but appears slow (S2 Video, S1 Fig).

### Health status of the animals

**Fig 3:**
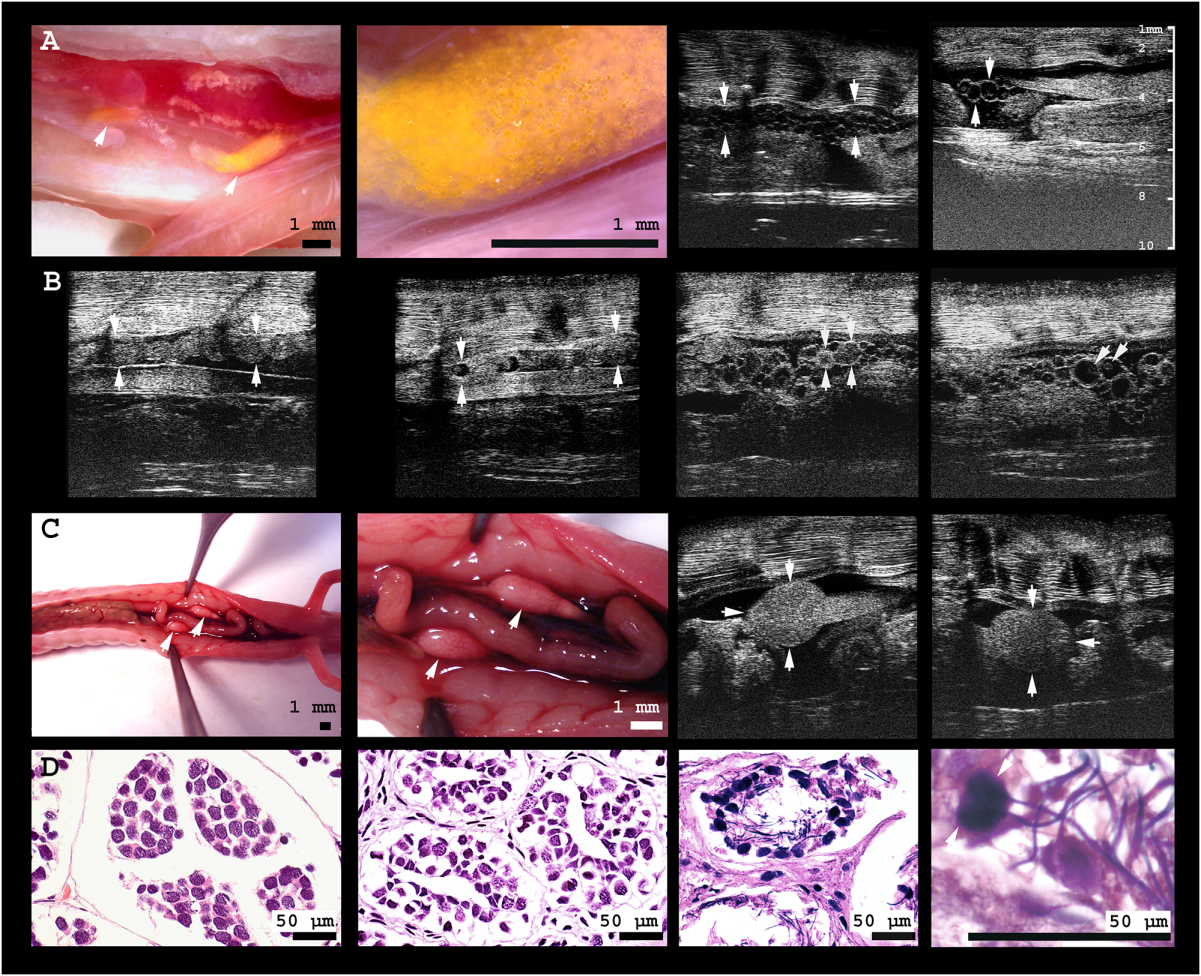
Gonads of male and female olms. **A** From left to right: (i) digital microscopic image of the caudal third of the body cavity of a female olm. The yellow pigmented ovary is located adjacent to the kidneys; the intestine is deviated to allow for free vision on the gonads. (ii) In close-up, small spherical follicles are visible within the translucent, comparably inactive ovary. (iii) Ultrasonographic image of an animal with follicles of small to medium size (≤ 0.5 mm), and of (iv) medium size (≤ 0.8 mm), including several homogenously echoic follicles; ovary margins are indicated by arrowheads. **B** From left to right: (i) The oviduct of the same individual without, and (ii) with oocytes inside; oviduct margins are indicated by arrowheads. (iii) Large oocytes of homogenous intermediate echogenicity, presumably representing sites of recently ovulated follicles (CLs) are indicated by the arrows, adjacent to (iv) comparably large (1.1 mm) vitellogenic follicles inside the ovary of the same individual; the yolk is visible as a slightly echogenic sphere within the anechogenic follicles. Note the large size differences of follicles within the ovary. **C** From left to right: (i) digital microscopic image of the caudal body cavity of a male olm. Testes are connected to and located cranially of the kidneys, adjacent to the intestine; (ii) close-up of the testes. (iii) Ultrasonographic image of left and (iv) right testis. **D** From left to right: (i) histological image of an individual with inactive testes, (ii) empty seminiferous tubules, and (iii) active spermatogenesis in the testes of another male olm; (iv) close-up of the testicular spermatozoa.

**Fig 4:**
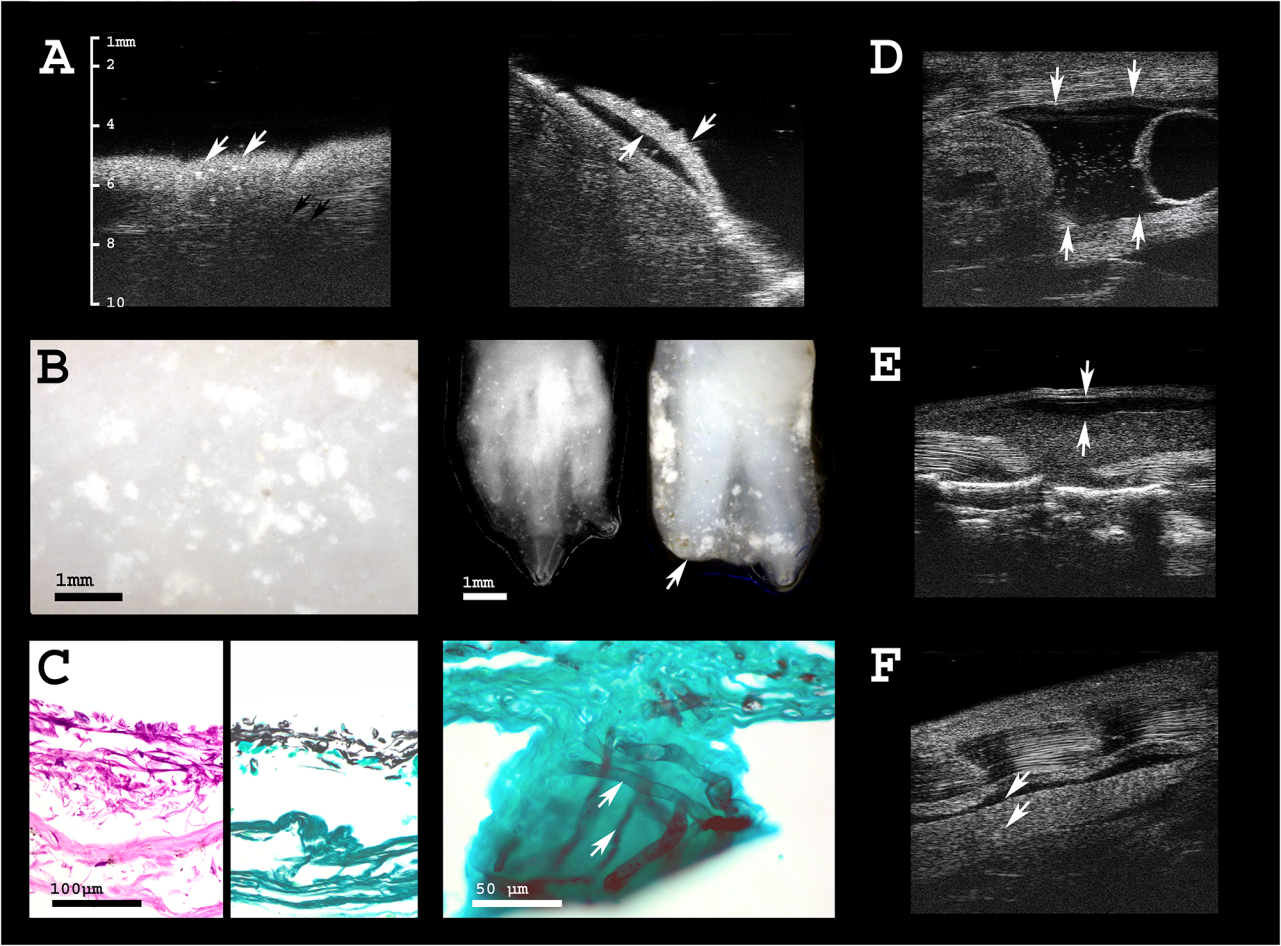
Health assessment of olms. **A** Ultrasonographic image of the skin of an olm that died due to *Saprolegnia* infection. White arrows indicate hyperechogenic foci in the skin that are not visible by adspection of the animal. Black arrows point to circular, hypoechogenic skin mucus glands (left), variably distributed across different body regions. Gills are severely damaged (right). **B** Digital microscopic images of the same individual; white spots indicate skin lesions caused by the fungal infection (left). Extremities are also severely affected; digits of both, three-toed hand and two-toed foot are partly destroyed by the infection, indicated by the arrow (right). **C** From left to right: histological sections of the infected skin, whose outer layers are detached and lost their integrity. Grocott stain of infected skin, and close-up to visualize fungal hyphae (stained in black) invading the epidermis. **D** Free coelomic fluid (physiological in amphibians; arrows) is augmented (ascites) and contains hyperechogenic particles in *Saprolegniasis*, indicating deteriorated health status. The bladder is seen on the right side of the image as echogenic membrane of 0.1–0.2 mm thickness, surrounding a circular space of anechogenic fluid at the caudal end of the body cavity. **E** One individual presented with subcutaneous edema, i.e. lymph accumulation within the dorsal lymph sac, with a wide range of possible underlying causes. **F** Two individuals showed sporadic hyperechogenic spots in the kidney that may indicate parasitic infection or renal deposits.

We ultrasonographically examined inner organs of eleven live and one dead olm, complemented by necropsy and histology of the latter (Figs 1, 3, and 4; for detailed organ appearance and measurements see S1 Fig and S1 Table). We measured several parameters that are relevant to health status and include: the functioning of the digestive system, food intake, body condition, and respiratory activity. Ultrasound also helped to detect several pathological alterations that allowed early diagnosis and treatment of fungal infection.

The anechogenic and spherical gall bladder is embedded within the proximal third of the liver (S1B Fig). In one of the live subjects, the gall bladder contained small amounts of hyperechogenic particles; the same individual displayed subcutaneous edema in the middorsal region (maximal distension 0.75 mm). Extensive hyperechogenic texture lined the gall bladder’s outer wall in the dead olm, likely representing a postmortem or freezing artifact due to crystallization of its content. Although they had been fed two weeks prior to the first, and only two days prior to the second ultrasound examination, the gallbladders of all individuals appeared relatively large and filled during both examinations. Mean gallbladder size was 7.1 ± 1.5 mm × 3.8 ± 0.7 mm at first assessment and 5.8 ± 1.0 mm × 3.4 ± 0.8 mm at second assessment (values represent maximal length in antero-posterior × height in dorso-ventral extension, assessed in sagittal plane). Assuming ellipsoid geometry, volume in the second compared to the first examination was 35% lower. This was statistically significant (p ≤ 0.005, V = 73 for length and p ≤ 0.016, V = 69 for height; paired Wilcoxon signed rank test).

In eleven of 13 animals, the lungs were clearly visible during ultrasound examinations (S1D Fig) and appeared to be of variable distensions, which were ranked in three categories (small, large, not detected). Occasionally, air-gasping behavior was observed in several, but not all individuals. Gill length was assessed in eight out of twelve animals (Fig 1A).

In four individuals, the kidneys displayed multiple small, markedly hyperechogenic foci.

Multiple disseminated and circumscribed hyperechogenic skin lesions (≤ 0.1 mm in diameter) were detected in the deceased, as well as in four of the live individuals (Fig 4A). Anechogenic fluid within the coelomic cavity was present in all of the animals that we examined, and was typically found at the caudal end of the body cavity, surrounding gonads and gut (Fig 4C). These anechogenic spaces appeared more extended in the individuals displaying skin lesions, and contained variable amounts of small dispersed hyperechogenic particles (Fig 4C).

### Sex determination and gonadal development

Sex determination is critical for captive breeding of olms, but gonads are not visible through the skin, nor is cloacal shape distinct between male and female individuals. Ultrasound allowed detection of testes in one of the twelve individuals. They were found cranially of, and connected to, the kidneys. They appeared to be of homogenous texture and intermediate echogenicity, located within the caudal third of the coelomic cavity (Fig 3C).

The eleven female olms showed different stages of ovarian development (Fig 3A and 3B) and were therefore of different value for captive breeding. Ovaries were irregularly shaped, containing clusters of follicles of various sizes surrounded by scarce amounts of slightly hyperechogenic tissue. Follicles were hypoechogenic, of spherical to oval shape, and surrounded by a thin hyperechogenic lining; maximal follicle diameter differed between individuals and ranged from 0.1–1.1 mm. In four females with intermediate follicle size (0.5–1.1 mm), a hyperechogenic and spherical central inner structure was visible. In one female, several spherical structures of homogenous intermediate echogenicity were visible in the ovary (Fig 3A) in addition to regular hypoechogenic follicles. This female was also the only one with a well-developed oviduct, visible as an undulating tissue strand in parallel to the ovaries and kidneys (0.9 mm in diameter). It contained several irregularly shaped oocytes with hyperechogenic content (diameter 0.8 mm, Fig 3B). In the remaining seven individuals with small (< 0.5 mm) follicles, the ooplasm appeared homogenously anechoic. Sizes of testes, follicles, and ovaries are given in S1 Table.

### Necropsy and histology

Necropsy was performed on one deceased female olm. Organ sizes obtained from scaled images correspond well with measurements obtained during the preceding ultrasound examination of the same individual (Table 2).

**Table 2:** Comparison of organ measurements by scaled digital images versus ultrasound during necropsy of a dead female *Proteus*.

Measurement	Scaled image or photograph	Ultrasonography
	diameter/length [mm]	diameter/length [mm]
**Body**	10.3 / 215*	- / -
**Coelomic cavity**	6.2 / 105*	- / -
**Heart**	3.1 / 9.7	3.3/ 9.7
**Liver**	4.3 / 75	4.1 / -
**Gall bladder**	3.4 / 5.2	3.0 / 5.4
**Lung**	2.7 / 81*	- / -
**Kidney**	1.8 / 36.2	1.6 / -
**Gut**	2.2 / 150*	2.0 / -
**Ovary**	1.2 / 22.3	1.2 / -

*measurements of large structures were performed on scaled digital photographs;

- measurement was not feasible by using ultrasound

To establish references for normal (S1 Fig) and pathologic organ appearance, the ultrasonographic aspect of inner organs was compared to the results of dissection and histology of this female individual (Figs 3, 4 and S1 Fig), and to histological testicular sections of two further male individuals (Fig 3D). The specimens available for this purpose were frozen several hours after death, which compromised histological quality; especially the delicate ovaries were largely autolytic and are therefore not shown. The only pathologically relevant lesions were detected in the deceased female’s skin. Grocott staining revealed the presence of fungal hyphae. In the liver, the conspicuous and abundant pigments stained partly positive for copper as indicated by Rhodamine, partly for lipofuscin as indicated by extended Ziehl-Neelsen staining, as well as massively for iron as indicated by Prussian Blue.

## Discussion

The majority of amphibian species have never been kept or bred in captivity; yet establishing husbandry and successful breeding programs [26,27] well ahead of acute population decline may be crucial to their conservation. Therefore, validated tools for monitoring health and assisted reproductive techniques are invaluable. Ultrasound offers great potential to fulfill this function in wildlife species [28,29], being non-invasive, innocuous, and safely repeatable [30]. It has been successfully applied in amphibians [31,32], which we here reaffirm for the endangered olm. Three valuable aspects can be addressed using this imaging modality: basic physiology, health monitoring, and reproductive monitoring.

### Basic physiology—Heart rate

Information obtained by ultrasound provides insights beyond purely medical assessment and diagnosis. Morphometry sheds light on development and physiology, while visualization of directional motion reflects vascular flow and heartbeat, parameters closely linked to metabolic activity [33]. Ultrasound frequencies between 4–12 MHz proved suitable for measuring heart rate in olms (42.9 ± 4.6 bpm, range 32–55 bpm). This is higher than the heart rate of 30 bpm reported for a *Proteus* larva (20.8 mm long), immediately upon hatching [14]. This finding is surprising, considering that freshly hatched larvae normally have substantially higher heart rates than adults [14, 34]. Heart rates of similarly sized amphibian species, at similar temperatures, are not reported in the literature. Heart rate may be overestimated in our setting due to increased activity or stress of handling. Although assessment of heart rate was feasible only when olms remained immobile for at least 10–20 s, most individuals displayed frequent movements in the examination chamber. Brief but intense activity has been shown to increase frog heart rates by up to 75% [34], whereas gentle handling alone seems to have little influence [35]. To examine heart rate during rest and explore its variability in relation to activity level may give interesting insight into their energy-saving strategies, especially when considering their reportedly low metabolic rate [36] and their astonishing ability to starve for up to several years [37]. Careful assessment will be needed, as heart rates of ectotherms depend on numerous variables and display large variations between taxa [38].

### Health monitoring

Diagnostic non-invasive imaging is invaluable for maintaining healthy captive and founder populations, which in turn are a prerequisite for establishing successful breeding programs. Ultrasound at frequencies of 50–70 MHz is capable of visualizing organs at almost microscopic detail in small organisms such as the olm. Detecting diseases at very early stages allows for timely treatment or for adjustments in husbandry before health problems become clinically relevant. Furthermore, individuals that exhibit pathologies compromising breeding or health of successive generations can be detected and excluded from reproduction. Morphology, anatomy, and organ appearance vary across taxa. To account for a lack in reference data, validation of ultrasound findings required postmortem anatomical and histological investigation of a deceased olm.

Ultrasonographic appearance of the inner organs was in general accordance with that of other amphibian species (S1 Fig). The liver showed high fat content in macroscopic, ultrasonographic, and histologic examinations. Large intracellular deposits of copper, lipofuscin, and haemosiderin were detected histologically. This has been described previously and linked to low metabolic rate, extended lifespan, and to the liver’s storage capacity, that buffers the scarce and infrequent food supply in their natural environment [10,39]. Inhomogeneities indicating pathology were not detected. Gall bladders were inconspicuous in all but one live subject. This animal’s gall bladder contained small amounts of hyperechogenic content. During both examinations, gall bladders were relatively large, filled, and approximately spherical. The shorter feeding interval preceding the second, compared to the first, examination (two days versus 14 days), was accompanied by an average volume reduction of 35%. Gall bladder size and content of stomach and intestines may serve as an indicator for nutritional status, functioning of the digestive system, and time-interval since last feeding, as demonstrated across various vertebrate taxa, e.g. humans [40], cats [41], opossum [42], chicken [43], and fish [44]. Generally, bile is released shortly after feeding and replenished slowly thereafter; extent, timing, and duration of these events are species-specific. Systematic longitudinal measurements at various intervals before and after feeding are needed to determine their normal sequence, possibly revealing interesting insight into the digestive system of a species capable of extreme long-term starvation. Monitoring gut content within the intestine (gut passage), and the rate of its decomposition may help to optimize husbandry. Food items were unrecognizable two days after feeding, indicating effective biochemical degradation, given that an olm’s teeth (Fig 2C) are used for capturing prey, but incapable of effective grinding [45]. The relatively short digestive tract approximates 1.4 times the length of the coelomic cavity (Table 2), consistent with a carnivorous life-style. Although its different sections, including the stomach, were indistinguishable both macroscopically and ultrasonographically, they can be differentiated in histological sections [46,47].

Variable distensions of the lungs were noted ultrasonographically. In two animals, the lungs were undetectable, thus probably devoid of air at the time of examination. Anatomy and function of the lungs have been discussed in detail by Oppel (1889), who confirmed their repeatedly questioned functionality and homology to regular vertebrate lungs [47]. The oral cavity and lungs of *Proteus* further serve as resonators [48] and—through a solid anatomical connection with the oval window of the inner ear—as an underwater pressure converter. Orientation of *Proteus* in complete darkness greatly depends on its ability to localize vibrations from prey and other sound sources; their underwater hearing is exceptionally developed, especially in the low frequency range [48]. If and to what extent the observed deflation of the lungs may compromise hearing remains to be tested. Whether lungs are used for respiration may depend on dissolved oxygen concentration, on the various factors influencing oxygen demand, and on the efficiency of oxygen uptake via skin and gills. Interestingly, the two individuals with severe lesions of skin and gills breathed more frequently, whereas air gasping behavior had not been noted in the individuals without detectable lungs. Gill length depends on oxygen availability [49,50] and values (4.6 ± 0.8 mm in a range of 3.3 mm to 5.9 mm; n = 8; S1 Table) were comparable to those given by Fitzinger (1850) (3.6–1.5 mm in a range of 1.6 mm to 6.2 mm; n = 7, [51]), indicating oxygenation of the aquaria to approximate natural conditions.

We detected several subclinical pathologies. In four olms, several disseminated, small, circumscribed echodense foci were detected in the kidneys, indicating renal deposits, parasites, or other infections (Fig 4F). Gout is unlikely to occur in aquatic amphibians, whereas parasitic infection has been described (*Chloromyxum protei*) [52]. No renal pathology was found in histology; however, sonographically, the above-mentioned renal lesions were not detected in the specimen that was used for histology. Skin lesions of the two deceased olms, associated with the loss of toes and gills, tested positive for *Saprolegnia spp*.. Besides apparent gross lesions, ultrasonographically, small circumscribed and highly echogenic foci were evident within the outer skin layers, invisible to the human eye (Fig 4). Such lesions, apparently representing subclinical stages of infection, were detected in four additional live olms. These tested positive for *Saprolegnia*, whereas all other olms tested negative. This helped to initiate early treatment and cure all but the most severely affected individual, which died from the infection. Specific microbiological findings and treatment is discussed in more detail elsewhere [53].

All animals showed moderate amounts of anechogenic fluid surrounding the organs within the caudal coelomic cavity. This is a physiological finding in amphibians [31] and should be distinguished from hydrocoelom (ascites). In the individuals most affected by skin lesions, coelomic fluid was more abundant and contained variable amounts of dispersed hyperechogenic particles (Fig 4D).

One olm presented with biliary sediment which can be caused by parasitic, infectious, or alimentary reasons. For example, in lizards, biliary sludge has been linked to a diet overly rich in lipids [54]. The same individual showed subcutaneous edema. Hydrocoelom and subcutaneous edema (ascites and anasarca) are not uncommon in amphibians; accumulation of fluid in the subcutaneous lymphatic sacs may be caused by lymph heart or cardiac failure, neoplasia, toxicosis, gastrointestinal, renal, or hepatic disease, infection, or improper environmental conditions. Further diagnostics, e.g. coeliocenthesis and cytology, microbiological and fungal culture [55] are needed (for an overview, see [56]).

In summary, inner organ appearance and health status was slightly compromised in five of the eleven live individuals. Besides these subclinical and possibly husbandry-related findings, the major health risk seems to be imposed by skin infections. Flushed animals seem to be at elevated risk of developing and dying from infectious diseases such as *Saprolgniasis* (i.e., all of the cases described here, including the fatal ones). So far, health status and threats of wild *Proteus* have not been studied.

### Reproductive monitoring—Sex and reproductive status

Sex determination based on external traits alone is difficult and unreliable in largely monomorphic species such as the olm. The same holds true for assessing gonadal activity and reproductive status. Ultrasonography non-invasively provides information on size and blood perfusion of gonads, sperm and egg production, maturation, and ovulation and may be complemented with measurements of hormone levels. Insight into reproductive cycles, seasonality, function, and senescence [57] is crucial for selection of suitable partners and optimal timing. Further, ultrasound may serve to monitor assisted reproductive techniques [58], e.g. the success of inducing spermiation and ovulation by exogenous hormones. As breeding of olms is extremely difficult and has been rarely achieved, these insights are invaluable for establishing successful captive breeding programs.

We confirm the common notion of monomorphy of female and male olms by external cues [16]. Sexually active males have been reported to have slightly larger cloacae than females, and gonads may shine through the unpigmented skin [14]. Nevertheless, given reproductive intervals of more than a decade, a reliable innocuous method for sex determination is crucial. Sex ratio of the captive olms in our sample was extremely biased: the only male was an individual captured from the wild (1 male, 4 females), while all animals flushed from the caves were female (0 males, 8 females). In amphibians, a variety of different mechanisms for sex determination exist and might explain such a bias (for a review, see [59]). It is unknown which mechanism determines sex in olms. Whether a generally biased sex ratio exists in olms—as reported for some mammals [60] and for many amphibians due to environmental factors [61] or pollutants [62]—a higher probability for females to be flushed from the caves or captured in the wild, or greater survival of females during suboptimal environmental conditions, remains highly speculative. Unfortunately, available data is insufficient to answer this and to rule out chance. Interestingly, in Herrmann’s cave, Germany, one of six caves to which olms have been artificially introduced, reproduction initially failed as all animals were male [63], rendering a general bias towards females improbable.

Oviparity is the common mode of reproduction in olms [14,15], despite questionable reports of ovoviviparity [64,65], possibly provoked by adverse husbandry conditions. According to body mass (14.2 ± 5.2 g; range 7.2 to 24.2 g) and fully developed extremities, all of the olms at Zagreb Zoo were adult, although not all may have reached sexual maturity. The female with the lowest body mass (7.2 g), had an inactive ovarian appearance and homogenous follicle diameters of ≤ 0.1 mm. All further females had slightly oval follicles of larger and variable size, indicating active oogenesis. In seven females, follicles measured less than 0.5 mm in diameter and appeared anechogenic, representing pre-vitellogenic stages [66], whereas in another four individuals with follicles between 0.5 and 1.1 mm, spherical echodense content was visible (vitellogenesis, compare [67]). The ovary of one female with follicle sizes of up to 1.1 mm displayed a clearly visible oviduct, containing oocytes in its medial part, as indicated by their shape, which is characteristic of deformation within the oviduct [68]. This finding is indicative of previous breeding activity and recent ovulation. These nevertheless appeared smaller (0.8 mm) than expected for oocytes ready for oviposition, were of irregular shape, and contained a hyperechogenic center. Assuming no further growth to occur inside of the oviduct, except for deposition of the jelly coat, they were likely undergoing resorption. In amphibians, resorption of oocytes may be triggered by stress [69]; indeed, health of the respective individual was compromised as it was affected by *Saprolegniasis*. The maximal follicle diameter recorded in this study is far smaller than the reported size of 4–5 mm for recently deposited eggs, which by swelling of the surrounding jelly coat a few hours after oviposition reach diameters of 8–9 mm [70]. None of the examined individuals seemed to contain mature oocytes, i.e. ready for oviposition. This is plausible, given the large intervals between successive breeding events of female olms.

It will be interesting to address the question of how male spermatogenesis and spermatophore production relates to female reproduction. The occurrence of three morphological forms of testes, probably related to gonadal maturation, has been described by Mali and Bulog (2015) in dead specimens [71]. We did not detect spermatophores deposited from previous interactions with males inside of the female genital tract. Spermatophores, however, may be difficult to visualize by ultrasound; also, they may have disintegrated during the period of solitary husbandry at Zagreb Zoo. Longitudinal monitoring of live olms of both sexes, comprising situations of active breeding, will be of great value to shed light on the dynamics, morphologic changes, and timing of olm reproduction.

We identified a potential breeding pair, choosing the only male by default, and the only female of the same region (Picinova cave). Geographic origin was considered, as a taxonomic division of olms into different subspecies has been previously proposed [50,51,72]. The pair has been housed in the same aquarium, but so far, no reproductive activity has been recorded.

### Conclusions and outlook

One third of all amphibians are currently listed as globally threatened or extinct [1], rendering them the most rapidly declining vertebrate class. Captive maintenance and breeding populations are a prerequisite for a potential future restoration of natural populations of endangered species. We show that ultrasound is a non-invasive, precise, and practical tool for health monitoring, sex determination, and assessment of reproductive status in olms, and possibly also in other salamander and amphibian species. Furthermore, indirect measures for physiological processes, such as heart rate, gall bladder size and functioning of the digestive, respiratory, and urogenital system can be observed. This may be helpful to optimize husbandry, detect disease such as fungal infections or parasites early, and initiate veterinary intervention in a timely manner. Longitudinal measurements of physiological and reproductive parameters, and a comparison to olms in the wild, will be of great interest. Assisted reproductive techniques, such as hormonal induction of ovulation and spermiation as well as artificial insemination may be the next useful steps towards efficient captive breeding of this species.

Beyond its value for captive maintenance and breeding, monitoring health status of wild populations will be of great value for conservation. Pathogens, such as fungal and bacterial skin infections may be facilitated by increasing ambient temperatures due to global warming. Early detection of such developments may help to initiate remedies in time.

## Supporting information

S1 Video Beating heart of *Proteus anguinus*. Blood flow appears orderly without major turbulences during each heartbeat. Images were acquired in color Doppler-mode at 12 MHz.
(ZIP)

S2 Video Blood flow within the dorsal aorta of *Proteus anguinus*. Adjacent to the gall bladder, blood flow is shown within the dorsal aorta, located between liver (a section of the gall bladder is visible at the right side of the image) and vertebral column. Compared to the liver, the content of this large vessel with apparently slowly moving content presented slightly hyperechogenic. Images were acquired in B-mode at 48 MHz.
(ZIP)

S1 Fig Inner organs of the olm. Ultrasonographic images are shown in the left column, corresponding digital microscope images and histological sections in the middle and right columns, respectively. **A** The heart in lateral view, located centrally within the body cavity, slightly cranial to the insertion of the fore-feet. The single ventricle and auricle appear with intermediate echogenicity in ultrasound; due to the moderate echogenicity of the large nucleated erythrocytes, the heart displays little contrast to the contained blood. It is enclosed by a pericardial sac containing anechogenic fluid. **B** The liver of olms is of almost spherical proportions, partly enclosing the intestine, and is characterized by homogeneous texture of intermediate echogenicity in ultrasound. The liver measures approximately one third of the olm’s total body length, and spans most of the viscera except for the heart and the upper part of the lungs, thinning out at the caudal end. The microscopic photograph of the liver shows the centrally located, comparably large gall bladder of greenish color, due to progressive autolysis. In histology, HE staining is shown on the left, and copper, lipofuscin, and iron staining (from top to bottom) on the right side. **C** The intestine stretches as a muscular tube along the entire body cavity, forming several loops. A distinction between large and small intestine, stomach or rectum is only evident in histological sections. **D** Cranially, the lungs begin with a short tracheobronchial portion and stretch as long, slim, air-filled sacs from near the pericardium towards the cranial end of the gonads. In ultrasound images they present as prominent hyperechogenic line with complete sound inhibition underneath. Macroscopically, lungs appear as long-stretched and air-filled vesicular structures. In histology, they present comparably dense, characterized by connective tissue and capillaries, due to collapsing of the air-filled cavities. The proximal airways (larynx) contain cartilage, shown on the right side of the histological image. **E** The paired kidneys are cigar-shaped, dark red structures within the caudal third of the body cavity, in proximity to the vertebral column. In females, the white and convoluted oviducts line their lower margins. In ultrasound, their texture appears coarser and slightly hyperechogenic compared to the liver. Glomeruli are visible in ultrasound and histological sections. **F** The body wall from a lateral transversal view. Two distinct skin layers, dermis and epidermis, are distinguishable in ultrasound. Dorsal muscles of approximately triangular shape span between the successive, echodense vertebrae. They present with intermediate echogenicity and thin interspersed hyperechogenic streaks. Macroscopically, on a skin flap, the outer, slightly pigmented skin surface is visible.
(TIF)

S1 Table Ultrasonographic measurements of *Proteus anguinus* organs. (DOCX)
