# Supplementary figures and images for: Monitoring health and reproductive status of olms (Proteus anguinus) by ultrasound
Source: PLoS One. 2017 Aug 15;12(8):e0182209. doi: 10.1371/journal.pone.0182209 (PMC5557490; doi:10.1371/journal.pone.0182209)

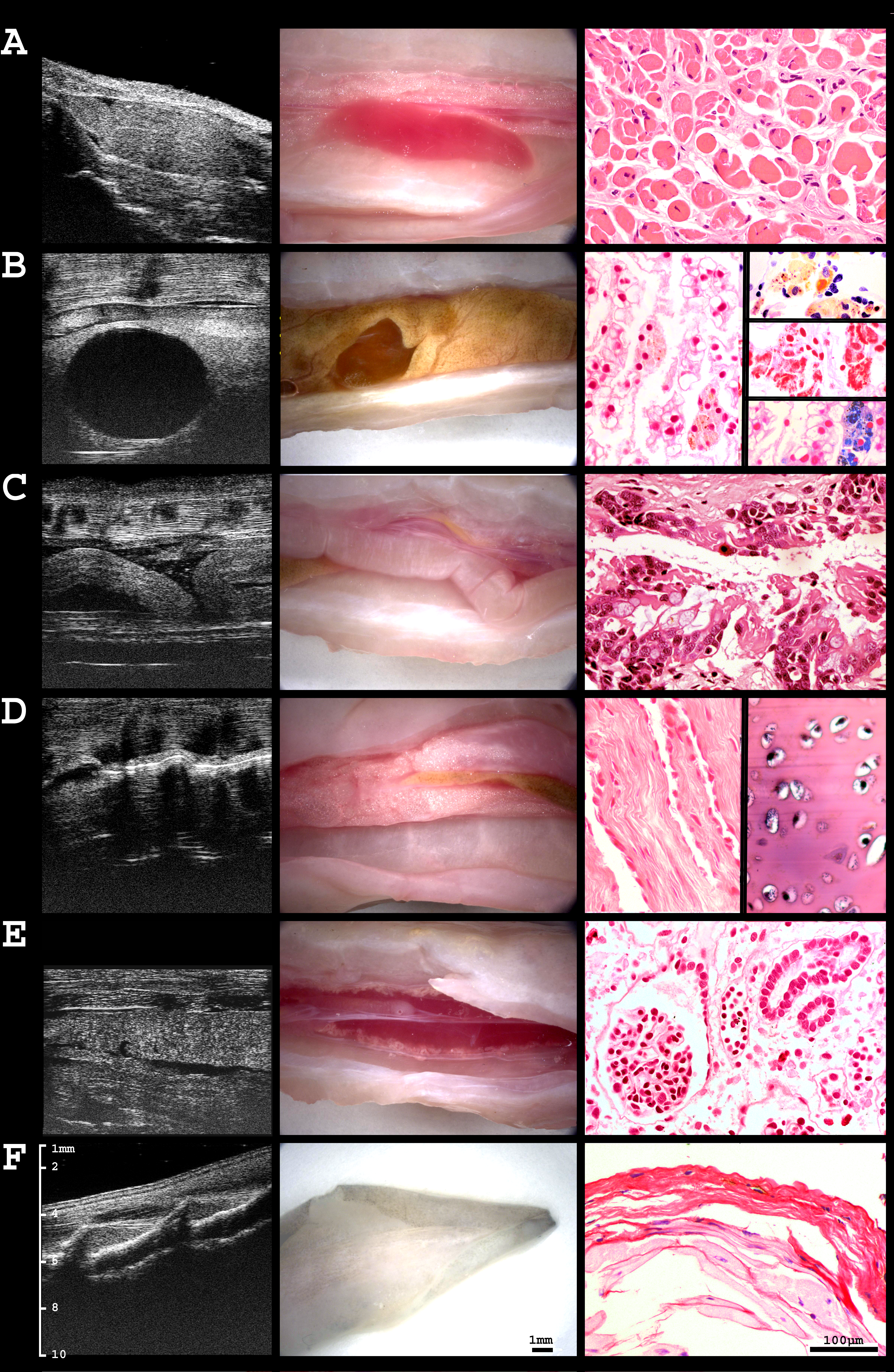

Supplement: S1 Fig — Ultrasonographic images are shown in the left column, corresponding digital microscope images and histological sections in the middle and right columns, respectively. A The heart in lateral view, located centrally within the body cavity, slightly cranial to the insertion of the fore-feet. The single ventricle and auricle appear with intermediate echogenicity in ultrasound; due to the moderate echogenicity of the large nucleated erythrocytes, the heart displays little contrast to the contained blood. It is enclosed by a pericardial sac containing anechogenic fluid. B The liver of olms is of almost spherical proportions, partly enclosing the intestine, and is characterized by homogeneous texture of intermediate echogenicity in ultrasound. The liver measures approximately one third of the olm’s total body length, and spans most of the viscera except for the heart and the upper part of the lungs, thinning out at the caudal end. The microscopic photograph of the liver shows the centrally located, comparably large gall bladder of greenish color, due to progressive autolysis. In histology, HE staining is shown on the left, and copper, lipofuscin, and iron staining (from top to bottom) on the right side. C The intestine stretches as a muscular tube along the entire body cavity, forming several loops. A distinction between large and small intestine, stomach or rectum is only evident in histological sections. D Cranially, the lungs begin with a short tracheobronchial portion and stretch as long, slim, air-filled sacs from near the pericardium towards the cranial end of the gonads. In ultrasound images they present as prominent hyperechogenic line with complete sound inhibition underneath. Macroscopically, lungs appear as long-stretched and air-filled vesicular structures. In histology, they present comparably dense, characterized by connective tissue and capillaries, due to collapsing of the air-filled cavities. The proximal airways (larynx) contain cartilage, shown on the ri [file pone.0182209.s003.tif]
