# Supplementary material for: Monitoring health and reproductive status of olms (Proteus anguinus) by ultrasound
Source: PLoS One. 2017 Aug 15;12(8):e0182209. doi: 10.1371/journal.pone.0182209 (PMC5557490; doi:10.1371/journal.pone.0182209)
Supplement: S1 Table — (DOCX) [file pone.0182209.s004.docx]

**S1 Table. Ultrasonographic measurements of *Proteus anguinus* organs.**

| **Measurement** | **Dimension** | **Unit** | **Average** | **Stdev** | **Min** | **Max** | **N** |
| --- | --- | --- | --- | --- | --- | --- | --- |
| **Body mass** | weight | [g] | 14.2 | 5.2 | 7.2 | 24.2 | 12 |
| **Vertebrae** | length | [mm] | 4.5 | 0.5 | 4.0 | 5.6 | 12 |
| **Gills** |  |  | 4.6 | 0.8 | 3.3 | 5.9 | 8 |
| **Heart*** | length × width | [mm × mm] | 9.4 × 3.0 | 1.6 × 0.6 | 6.0 × 2.0 | 12.1 × 4.1 | 13 |
| **Liver** | diameter | [mm] | 4.4 | 0.7 | 3.5 | 5.4 | 12 |
| **Aorta** |  |  | 1.0 | 0.1 | 0.8 | 1.2 | 12 |
| **Gut** |  |  | 1.8 | 0.1 | 1.5 | 2.0 | 12 |
| **Kidney** |  |  | 1.6 | 0.3 | 1.2 | 2.1 | 12 |
| **Glomeruli** |  |  | 0.5 | 0.1 | 0.4 | 0.7 | 12 |
| **Bladder** |  |  | 3.0 | 0.7 | 2.4 | 4.0 | 4 |
| **Dorsal muscles** |  |  | 3.6 | 0.3 | 3.1 | 4.0 | 12 |
| **Epidermis** |  |  | 0.2 | 0.0 | 0.1 | 0.2 | 12 |
| **Dermis** |  |  | 0.2 | 0.0 | 0.1 | 0.3 | 12 |
| **Skin glands** |  |  | 0.3 | 0.0 | 0.2 | 0.3 | 12 |
| **Ovary** |  |  | 1.3 | 0.3 | 0.9 | 1.5 | 11 |
| **Follicles (max.)** | length × width | [mm × mm] | 0.5 × 0.4 | 0.3 × 0.3 | 0.1 × 0.1 | 1.1 × 1.0 | 11 |
| **Testis*** |  |  | 3.7 × 2.2 | 0.2 × 0.2 | 3.5 × 2.0 | 3.9 × 2.4 | 2 |
| **Eye** |  |  | 1.5 × 0.5 | 0.5 × 0.0 | 1.1 × 0.5 | 1.8 × 0.5 | 2 |
| **Ear** |  |  | 0.9 × 0.7 | 0.2 × 0.3 | 0.6 × 0.4 | 1.2 × 1.1 | 6 |
| **Brain*** |  |  | 5.9 × 2.0 | 0.6 × 0.6 | 5.5 × 1.5 | 6.8 × 2.9 | 4 |
| **Dors. lymph heart** |  |  | 0.5 × 0.5 | 0.1 × 0.1 | 0.5 × 0.4 | 0.7 × 0.6 | 3 |

* craniocaudal × dorsoventral dimension, measured in sagittal view
